# Toxic Metals in Surface Dust in Underground Parking Garages: Pollution Status, Risk and Disease Burden Assessment, and Source Apportionment

**DOI:** 10.3390/toxics13100895

**Published:** 2025-10-19

**Authors:** Yong Wang, Tong Chao, Qidi Li, Zhiqiang Jiao, Xinling Ruan, Yuguang Wang, Shiji Ge, Yangyang Wang

**Affiliations:** 1 School of Material and Chemical Engineering, Tongren University, Tongren 554300, China; wy7185299@126.com; 2 College of Geographical Sciences, Faculty of Geographical Science and Engineering, Henan University, Zhengzhou 450046, China; chaotong1229@henu.edu.cn (T.C.); liqidi9821@henu.edu.cn (Q.L.); zqjiao@henu.edu.cn (Z.J.); xlruan@henu.edu.cn (X.R.); 3 Key Laboratory of Geospatial Technology for the Middle and Lower Yellow River Regions, Ministry of Education, Henan University, Kaifeng 475004, China; 4 School of Minerals Processing and Bioengineering, Central South University, Changsha 410083, China; ygwang@csu.edu.cn

**Keywords:** urban built environment, dust contamination, disability-adjusted life years, APCS-MLR model

## Abstract

Surface dust serves as a significant carrier and potential source of various pollutants in urban environments. However, limited attention has been paid to toxic metals in underground parking garages’ (UPGs) surface dust. In this study, thirty surface dust samples were collected from UPGs to determine the toxic metals contents, their risk and disease burden to local residents, and their potential source. The mean contents of V, Cr, Co, Ni, Cu, Zn, Cd, Sb, Pb, Hg, and As were 68.06, 126.48, 8.73, 27.68, 76.25, 287.07, 0.74, 4.28, 172.67, 0.24, and 8.66 mg/kg, respectively. Accumulation index revealed that the geoaccumulation index of Cr, Cu, Cd, Zn, Sb, Pb, and Hg ranged from 0.52 to 1.85. Pollution load index verified that the surface dust was slightly (56.67%), moderately (30.00%), or heavily polluted (13.33%). Risk assessment revealed that the total non-carcinogenic risks for children all exceeded the acceptable level (HI > 1.0). Notably, the carcinogenic burden reached 12.9 disability-adjusted life years per 100,000 population, with Cr contributing 84.1%. Furthermore, these toxic metals mainly derived from vehicle-related activities, use of coal, and the aging of decoration materials, and their accumulation in UPGs’ surface dust was almost unaffected by the essential conditions of residential areas.

## 1. Introduction

Rapid industrial and economic development has led to a substantial increase in the number of automobiles in China, from 3.5 million in 1990 to 450 million in 2024. This increase has resulted in a shortage of parking spaces, especially in the densely populated urban areas [1]. To address this issue, numerous underground parking garages (UPGs) have been constructed and are becoming indispensable infrastructure in modern cities [2]. Although UPGs effectively alleviated the shortage of parking spaces, their potential threats to human health also warrant considerable attention. Most UPGs in China are semi-closed, the limited air exchange and untimely diffusion can result in the accumulation of pollutants in UPGs. In addition, UPG staff typically work approximately 4 h per day [1], some residents rest or engage in recreational activities in UPGs due to its characteristics of warm in winter and cool in summer, and some disadvantaged individuals even reside in these spaces [2]. Therefore, pollutants in UPGs may pose unexpected health risk to these staff and residents.

Surface dust in urban environment is recognized as an important carrier and potential source of various pollutants and its impact on human health has been extensively studied [3]. Tarafdar et al. [4] and Cao et al. [5] have verified that the surface dust in hotels and rubber playgrounds contain high content of polycyclic aromatic hydrocarbons (PAHs), posing serious risk to human health. Similarly, the toxic metal contents in surface dust in factories and smelters were significantly higher than that in street dust, presenting a serious threat to surrounding residents [6,7]. Previous studies have also examined the concentrations and potential risks of various pollutants in the surface dust in driving schools, parks, playgrounds and even indoor environments [4,5,8,9]. However, the attention paid to the surface dust in UPGs is insufficient, and no reports regarding toxic metals in UPGs surface dust and their potential risk have been conducted.

Toxic metals are ubiquitous in various environments and can enter UPG environments through multiple pathways. Friction between tires and roads can results in the release of Zn and Cu [7], and the burning of fossil fuel can release Pb, Ni, Hg, and As [10,11]. The natural aging of fire-retardant and ground coatings used in UPGs may also release toxic metals [12]. These toxic metals often adhere to particles and then are deposited onto the ground [13]. However, these dust particles (especially fine particles) can be re-suspended and then enter into the human body via ingestion, inhalation, and dermal absorption [14]. Due to the persistence and non-biodegradability of these toxic metals, they can evade metabolic clearance and accumulate in specific tissues and organs [15]. For instance, Cr can cross the biological membranes and accumulate in the lungs, liver, and kidneys; Pb tends to deposit in bones and the central nervous system; and Hg can accumulate in the brain and thyroid gland [16,17]. Chronic exposure to these toxic metals, even at low levels, can disrupt normal physiological functions and induce various diseases [18,19]. Therefore, investigating the pollution status, potential risk and disease burden of toxic metals in UPGs’ surface dust could provide significant suggestions for the people who stay in UPGs for a long time.

The primary objectives of this study were to: (1) determine the contents and pollution status of V, Cr, Co, Ni, Cu, Zn, Cd, Sb, Pb, Hg, and As in UPGs surface dust; (2) estimate the potential health risks and carcinogenic disease burden caused by these toxic metals; (3) identify their possible sources by an absolute principal component score-multiple linear regressions (APCS-MLR) model.

## 2. Materials and Methods

### 2.1. Study Area

**Figure 1:**
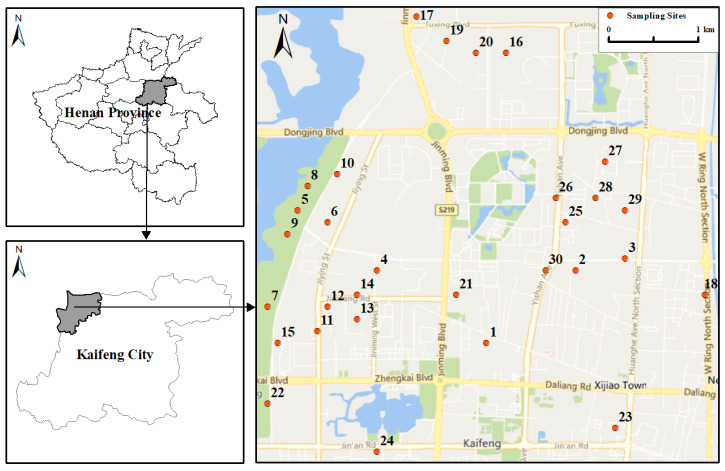
The location of Kaifeng City, study area, and sampling sites.

The study area was in the West Suburb of Kaifeng City, Henan Province, China (34°8′–35°4′ N and 114°35′–114°85′ E) (Figure 1). Kaifeng experiences a typical temperate continental monsoon, with an average annual temperature of 14.52 °C and precipitation of 640 mm. Kaifeng is a renowned tourist city with the tertiary industry as the primary source of the local economy, and its environmental quality is relatively good. The total administrative area of Kaifeng is 6116 km^2^, with an urban area of 803 km^2^. The total population of Kaifeng is approximately 4.70 million, including approximately 1.10 million in the Urban District and 0.15 million in the study area. The number of vehicles in Kaifeng City reached 862.3 thousand in 2024.

### 2.2. Sample Collection

A total of thirty surface dust samples (labeled 1 to 30) were collected from different UPGs based on their location and essential conditions (Figure 1). To minimize heterogeneity and uncertainty, each sample was composed of ten to fifteen subsamples at each UPG. Subsamples were obtained by sweeping surface dust from a 1.0 m^2^ area, and these subsamples were mixed and placed in paper envelopes, then transported to the laboratory. These samples were air-dried and manually crushed to pass through the 60- and 100-mesh nylon sieve for toxic metals analysis.

To investigate the impact of residential area essential conditions on accumulation of toxic metals in UPGs surface dust, 30 survey questionnaires (one per residential area) were conducted to collect the information related to the basic characteristics, usage, and maintenance of the UPGs. The collected information includes the completion time of the residential area, the coverage rate of green land, the size of the residential area, the occupancy rate, the property management fee, the number of total parking spaces, the utilization rate of these parking spaces, and the cleaning frequency. The detailed questionnaire is provided in the Supplementary Materials (Text S1 and Table S1). The relationships between these essential conditions and toxic metals pollution status in UPGs’ surface dust were analyzed using Pearson correlation analysis (origin 8.5, OriginLab, Northampton, MD, USA).

### 2.3. Toxic Metal Analysis

The As content in surface dust was determined by Atomic Fluorescence Spectrometry (AFS-3100, Beijing Haiguang Instrument Company, Beijing, China) after being digested by mixed hydrochloric acid and nitric acid (3:1, *v*/*v*), with a method detection limit (DL) of 0.3 μg/L. The Hg content was measured by cold vapor generation-atomic fluorescence detection (DMA-80, MileStone, Milan, Italy), with a DL of 0.05 μg/kg. The contents of V, Cr, Co, Ni, Cu, Zn, Cd, Sb, and Pb were measured by inductively coupled plasma mass spectrometry (ICP-MS, Thermo Fisher X2, Waltham, MA, USA) after being digested by HNO_3_-HF-HClO_4_, with DLs ranging from 0.03 to 0.15 μg/L (Table S2). The standard substance (GSS-17, Chinese Academy of Geological Sciences, Shijiazhuang, China) and blank was used for quality assurance, with the recoveries all within the acceptable range (95–105%). All analyses were conducted in triplicate, with the relative standard deviations ranging from 2% to 4%.

### 2.4. Contamination Assessment

The geoaccumulation index (*I_geo_*) was used to evaluate the pollution status of individual toxic metals in the surface dust, which can be calculated as follows [20]:

(1) $$I_{geo}=\log_{2}\frac{C_{i}}{{kB}_{i}}$$

where *k* is constant (generally 1.5); *C_i_* represents the individual toxic metal content in surface dust (mg/kg), *B_i_* represents the environmental background value of these metals [21]. The *I_geo_* is divided into seven levels and the details are provided in Table S2 [22].

The pollution load index (PLI) was further used to assess the comprehensive pollution status of these toxic metals [23]. The *PLI* can be calculated based on the following equation:

(2) $$PLI=\sqrt[{n}]{\prod_{i=1}^{n}\frac{C_{i}}{B_{i}}}$$

where *C_i_* represents the individual toxic metal content in surface dust (mg/kg), and *B_i_* represents the environmental background value of these metals. The classification of PLI is listed in Table S3 [24].

### 2.5. Potential Risk Assessment

To quantify the health risks of toxic metals in UPGs’ surface dust, the average daily dose (ADD) via three key exposure pathways (direct ingestion, dermal absorption, and inhalation) was calculated [23]. The ADD for each pathway was calculated using the following equations:

(3) $$ADD_{ing}=\frac{CS\times IR_{ing}\times EF\times ED}{BW\times AT}\times 10^{-6}$$

(4) $$\mathrm{AD}D_{\mathrm{der}}=\frac{\mathrm{CS}\times \mathrm{SA}\times \mathrm{AF}\times \mathrm{ABS}\times \mathrm{EF}\times \mathrm{ED}}{\mathrm{BW}\times \mathrm{AT}}\times 10^{-6}$$

(5) $$\mathrm{AD}D_{\mathrm{inh}}=\frac{\mathrm{CS}\times (1/\mathrm{PEF})\times IR_{\mathrm{inh}}\times \mathrm{EF}\times \mathrm{ED}}{\mathrm{BW}\times \mathrm{AT}}$$

where ADD_ing_, ADD_der_, and ADD_inh_ mg/(kg·day) are the average daily dose through direct ingestion, dermal absorption, and inhalation, respectively. CS is toxic metal content in UPGs’ surface dust (mg/kg). The meanings and detailed values of other parameters are listed in Table 1.

**Table 1:** The meanings and values of different parameters.

Exposure Parameters	Meaning	Unit	Children	Adult	Reference
BW	Body weight	kg	15	61.5	[25]
EF	Exposure frequency	d/a	180	180	[25]
ED	Exposure duration	a	6	24	[26]
IRinh	Inhalation rate	m^3^/d	10	20	[27]
IRing	Dust ingestion rate	mg/d	200	100	[26]
SA	Dermal exposure area	cm^2^	2800	5700	[26]
AF	Dermal adherence factor	mg/cm^2^	0.2	0.07	[26]
ABS	Dermal adsorption fraction		0.13	0.13	[26]
AT	Averaging life span	d	70 × 365	70 × 365	[28]
PEF	Particle emission factor	M^3^/kg	1.36 × 10^9^	1.36 × 10^9^	[25]

The considered metals all have chronic non-carcinogenic health risks to human, and can be assessed by target hazard quotient (HQ) and hazard index (HI) as follow equations [29]:

(6) $$HQ_{ij}=\frac{\mathrm{AD}D_{ij}}{\mathrm{Rf}D_{i}}$$

(7) $$\mathrm{HI}=\sum_{i=1}^{11}\sum_{j=1}^{3}HQ_{ij}$$

where ADD*_ij_* is the exposure of different exposure pathways for different toxic metals, mg/(kg∙d); RfD*_i_* is the reference dose mg/(kg·d) of toxic metal *i* [30], which is the largest amount of unit body weight that does not cause adverse reactions; HQ*_ij_* represent the single risk index (risk quotient) of toxic metal *i* through *j* exposure route; HI is the total non-carcinogenic health risk index of these toxic metals through three exposure routes. The HI or HQ ≤ 1 suggests that there is no probable risk of non-carcinogens. The value of HI or HQ > 1 means it has adverse effects on human health; the higher the value, the greater the health risk [16].

### 2.6. Disease Burden Assessment

Based on the epidemiological studies in Chinese populations, lung cancer was selected as the indicator of health outcome. The carcinogenic risk assessment was conducted according to the World Health Organization (WHO) methodology (WHO, 2021), quantifying the dose–response relationships and calculating the associated disease burden using carcinogenic slope factors (CSF) [31]. The CSF was calculated based on site-specific cancer incidence rates:

(8) $$CSF_{scaled,ep}=CSF\times \frac{Inc_{ep}}{Inc_{total}}$$

*CSF_scaled,ep_* is the scaled carcinogenic slope factor adjusted for the specific cancer site; *CSF* denotes the carcinogenic slope factor of toxic metals; *Inc_ep_* represents the incidence rate of the specific cancer type (e.g., lung cancer); *Inc_total_* refers to the overall incidence rate of all cancer types combined.

Carcinogenic risk (CR) represents the probability of cancer development following sustained lifetime exposure to a potential carcinogen. The calculation formula is as follows:

(9) CR=ADD×CSF

(10) $$P_{x}=CR\times \frac{RS_{x}}{S_{P}}$$

where *P_x_* denotes the age-specific annual cancer incidence rate. The *CR*, derived from the dose–response relationship, was converted to an annualized risk to enable cross-temporal comparisons [32,33]. *RS_x_* is the age-specific relative sensitivity estimated by dividing the age-specific cancer incidence rates by the total incidence rate, and *S_p_* is age span for each age group (4 for 0–4 age group and 5 for others).

The cancer burden was assessed using Disability-Adjusted Life Years (DALYs) as described by Pan et al. [34]. The DALYs comprised the years of life lost (YLLs) and the years lived with disability (YLDs). According to the disease model, DALYs, YLLs, and YLDs were calculated as follows:

(11) $$\mathrm{YLLs}=\sum_{x}n_{x}P_{x}(1-S_{x})(e_{x}^{*}-T_{D})$$

(12) $$\mathrm{YLDs}=\sum_{x,y}n_{x}P_{x}[(1-S_{x})DW_{y}L_{y}+S_{x}(DW_{y}L_{y}+P_{seq}DW_{seq}(e_{x}^{*}-T_{C}))]$$

(13) DALYs=YLLs+YLDs

where *n* denotes the population size; e* represents the standard life expectancy (from Global Burden of Disease); *L* indicates duration of disease state; *x* corresponds to age group; *y* identifies disease phase (e.g., diagnosis, treatment); *S_x_* signifies age-specific survival rate; *T_C_* and *T_D_* represent cure duration and time to death, respectively [35]; DW represents the disability weights for each cancer phase and is obtained from the Global Burden of Disease.

### 2.7. Source Appointment

The APCS-MLR is a receptor model that combines the statistical methods of principal component analysis (PCA) and multiple linear regressions (MLR). This model can qualitatively determine the load of each metal for each pollution source, and it also can quantitatively estimate the average contribution rate of the source to toxic metals and the contribution rate at each sampling site. The detailed calculation procedure followed the description of Ma et al. [36].

## 3. Results and Discussion

### 3.1. Toxic Metal Contents in UPGs Surface Dust

The toxic metal contents in UPGs’ surface dust are shown in Table 2. The mean contents of V, Cr, Co, Ni, Cu, Zn, Cd, Sb, Pb, Hg, and As were 68.06, 126.48, 8.73, 27.68, 76.25, 287.07, 0.74, 4.28, 172.67, 0.24, and 8.66 mg/kg, respectively, which were 0.81, 2.72, 0.90, 1.06, 3.71, 3.72, 2.46, 3.37, 7.02, 10.95, and 1.08 times higher than that of the background value in Kaifeng City [21,37]. The standard-exceeding ratio of these metals in UPGs surface dust relative to background value were 20%, 100%, 33%, 47%, 100%, 100%, 100%, 100%, 97%, 93%, 93%, and 40%, respectively. These results indicate that anthropogenic activities and natural aging of facilities have significantly increased the toxic metal contents in UPGs surface dust.

The coefficient of variation (CV) of Pb and Hg were 117.03% and 112.19%, respectively, indicating strong variation [38]. This result can be attributed to the use of Pb- and Hg-containing paints in some UPGs’ floors. In addition, the CVs of Cr, Cu, Zn, Cd, Sb, and As were all higher than 50% (high variability), indicating these toxic metals in UPGs’ surface dust may be strongly influenced by anthropogenic activities. The CVs of V and Co were 49.50% and 24.55% (moderate variability), respectively. Meanwhile, Ni exhibited the lowest variability (18.98%), indicating minimal influence from anthropogenic activities. Correlation analysis demonstrated significantly positive correlations between Cr and Cd, Cr and Ni, Cr and Hg, and Cd and Ni (Figure S1, *p* < 0.05), indicating these metals may come from the same pollution source. In addition, Co and Zn, Co and Sb, Co and As, Zn and Sb, Zn and As, Sb and As were significantly positively correlated (*p* < 0.05), indicating these metals may originate from another pollution source. The correlation between V, Cu, and Pb with other metals was non-significant (*p* > 0.05), suggesting these metals may originate from different sources with other toxic metals.

The contents of V in these UPGs’ surface dusts were similar to that of the surface dusts in bus stops [39] and parks [7] in Kaifeng, which further indicates that V was just slightly influenced by anthropogenic activities or specific pollution sources. However, the contents of Ni and Zn in these UPG surface dusts were lower than that in campus surface dust in Kaifeng (Table S4) [40]. In contrast, the contents of Cr, Pb, and Cu in these UPG surface dusts were significantly higher than that of other study areas (such as the bus stops, parks and campuses). This result implies that frequent access, starting and stopping, idling and braking of vehicles may be the primary source of Cr, Pb, and Cu in these UPG surface dusts.

### 3.2. Pollution Status of Toxic Metals in UPGs Surface Dust

**Figure 2:**
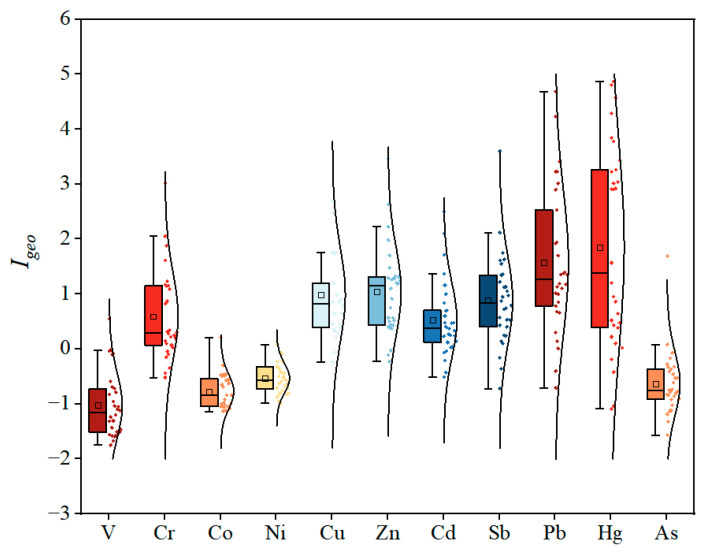
The *I_geo_* values of toxic metals in UPGs surface dust.

The *I_geo_* values of toxic metals in UPGs surface dust ranged from −1.75 to 0.55 for V, −0.52 to 3.02 for Cr, −1.14 to 0.21 for Co, −0.98 to 0.08 for Ni, −0.24 to 3.71 for Cu, −0.23 to 3.46 for Zn, −0.51 to 2.50 for Cd, −0.72 to 3.60 for Sb, −0.71 to 4.68 for Pb, −1.09 to 4.87 for Hg, and −1.57 to 1.69 for As, with the mean *I_geo_* values of −1.03, 0.59, −0.78, −0.53, 0.98, 1.05, 0.52, 0.88, 1.57, 1.85, and −0.63, respectively (Figure 2). The mean *I_geo_* values of V, Co, Ni, and As were lower than 0, indicating these UPGs surface dusts were unpolluted by these metals. The *I_geo_* values of Cr, Ni, Cu, Cd, and Sb ranged from 0 to 1 (unpolluted to moderately polluted), while the *I_geo_* values of Zn, Pb, and Hg ranged from 1 to 2 (moderately polluted). These results indicate that the accumulation of individual toxic metals in UPGs’ surface dust was moderate.

**Figure 3:**
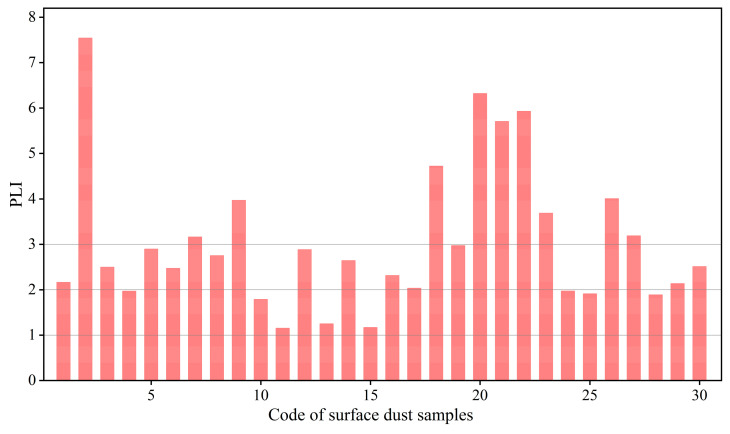
Comprehensive pollution analysis of toxic metals in UPGs surface dust.

The comprehensive pollution analysis showed that the PLI of these UPGs’ surface dust ranged from 1.11 to 4.18, with an average PLI value of 2.12 (Figure 3). All PLI values were higher than 1, indicating these UPGs surface dusts were polluted with toxic metals. Fortunately, more than 56.7% of these surface dust just slightly polluted (1 < PLI ≤ 2), while 30.0% were moderately polluted and 13.3% were heavily polluted. These results highlight that some UPGs’ surface dust is seriously polluted by toxic metals. In fact, toxic metal contamination in surface duct in various environments has been widely reported [17,41]. However, little attention has been paid to the toxic metals in UPGs surface dust. These findings underscore the urgent need to monitor the toxic metals in UPGs’ surface dust.

### 3.3. Non-Carcinogenic Risks Assessment

**Figure 4:**
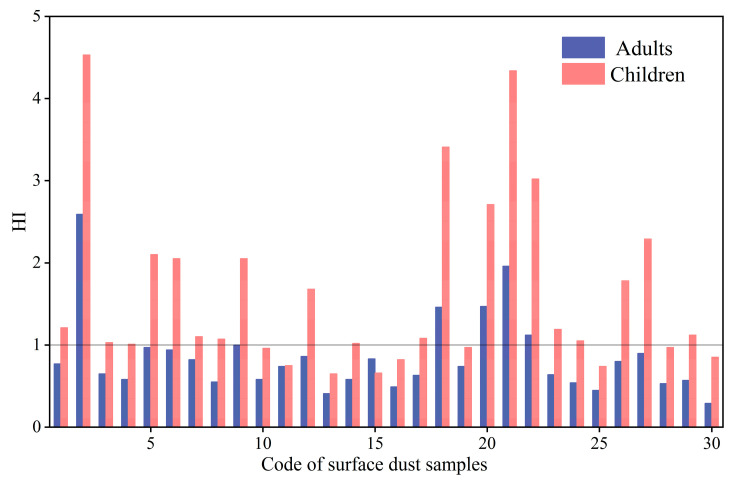
The non-carcinogenic risk of toxic metals in UPGs surface dust in different residential areas.

The non-carcinogenic risks via exposure to toxic metals in UPGs’ surface dust are shown in Figure 4. The results showed that the HI ranged from 0.29 to 2.59 for adults and from 0.65 to 4.53 for children, with the mean HI of 0.85 and 1.61, respectively. This result indicates that toxic metals in UPGs’ surface dust posed serious noncancerous disease risk to children, but their risks posed to adults are within the acceptable limits. In addition, the mean HI for children was 1.89 times higher than that of the adults, which can be attributed to the hand-to-mouth activity and the low tolerance level of children [22,42]. Furthermore, the mean HI value cannot fully represent the potential health risk of toxic metals in UPGs’ surface dust to residents in an individual residential area. For specific residential areas, the HI exceeding the acceptable level for adults and children accounted for 20% and 70%, respectively. These findings suggest that the potential health risks of toxic metals in UPGs’ surface dust in different residential areas varied obviously, but the reasons for the difference still needs to be further studied.

**Figure 5:**
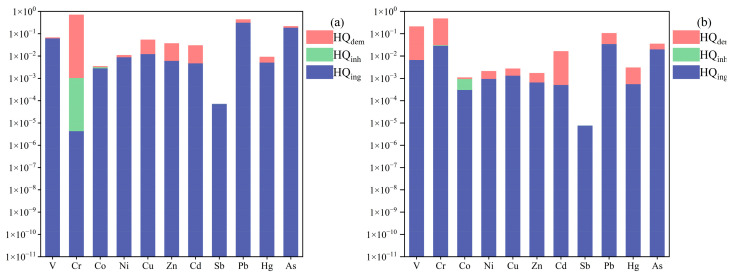
The contribution to HI of individual toxic metal through different exposure routes. (**a**): child; (**b**): adult.

The contribution of different exposure pathways to HI follows the following order: direct ingestion > dermal absorption > inhalation. The direct ingestion contributed 88.43% and 60.12% of the HI for children and adults, respectively (Figure 5), which is consistent with most previous studies [43]. For individual toxic metals, Cr contributed the most to HI, accounting for 43.46% and 55.92% for children and adults, respectively. Besides that, Pb, V, and As were also main contributors to HI for both children and adults, while other toxic metals had limited impacts. The HI of toxic metals in UPGs’ surface dust was relatively higher than that of the surface dust in parks [7] (HI: 0.13~0.18) and bus stops [39] (HI: 0.03~0.28) in the same study area, which can be attributed to the airtight and poor ventilation of UPGs (which hindered the spread and dilution of pollutants). These results further verified that the toxic metals in UPGs surface dust need more attention.

### 3.4. Disease Burden Assessment

The DALYs was used to assess the disease burden induced by Cr, Ni, Cd, Pb, and As for adults and children via three exposure pathways (Table 3). The result showed that the total population health burden was 12.9 DALYs per 100,000 population via toxic metals exposure in UPGs’ surface dust. Among them, the contribution of Cr was the highest (84.10%), followed by Ni (7.97%). The disease burdens attributable to As, Cd, and Pb were relatively low, collectively accounting for less than 8% of the total DALYs. These results demonstrate that the disease burden posed by individual toxic metals varied considerably, with Cr being the primary contributor to lung cancer burden.

Moreover, dermal contact was the main pathway for Cr-induced disease burden, accounting for 74.90% of Cr-specific DALYs and 62.99% of the total disease burden across all metals and pathways. Oral ingestion was the primary pathway for health burdens induced by Ni, Cd, Pb, and As. In contrast, inhalation exposure contributed minimally to DALYs across all metals, representing less than 0.3% of the total burden. This finding indicates that dermal contact represents the predominant exposure pathway for Cr-associated disease burden.

**Figure 6:**
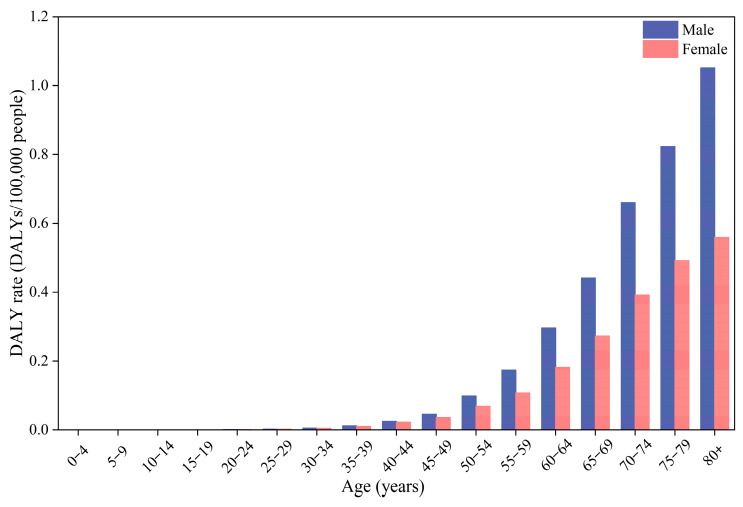
DALYs attributable to toxic metals in surface dust across age groups and sex.

In addition, the DALYs for males were significantly higher than for females across all metals and exposure pathways, with the disease burden of males being 1.70 times higher than that of females. Notably, the DALY rate (DALYs/100,000 people) displayed a distinct age-specific pattern, remaining low during infancy, childhood, and young adulthood, increasing significantly after the age of 50, and peaking in the 80+ age group (Figure 6). The elderly are more susceptible to carcinogenic effects from toxic metals, which can be primarily attributed to their declined immune function. Overall, males demonstrated a significantly higher disease burden than females across all age groups, with this sex-based disparity increasing with age.

### 3.5. Source Apportionment

Principal component analysis showed that three principal components with the eigenvalue higher than 1 were extracted, which could explain 77.94% of the total variables (Table S5). The first principal component explained 43.05% of the total variance and displayed strong loadings (>0.70) for Co, Zn, Sb, and As [41]. The second principal component accounted for 21.69% of the total variance and demonstrated strong loadings for Cr, Ni, Cd, and Hg. The third principal component explained 11.78% of the total variance and displayed strong loadings for Cu and Pb.

**Figure 7:**
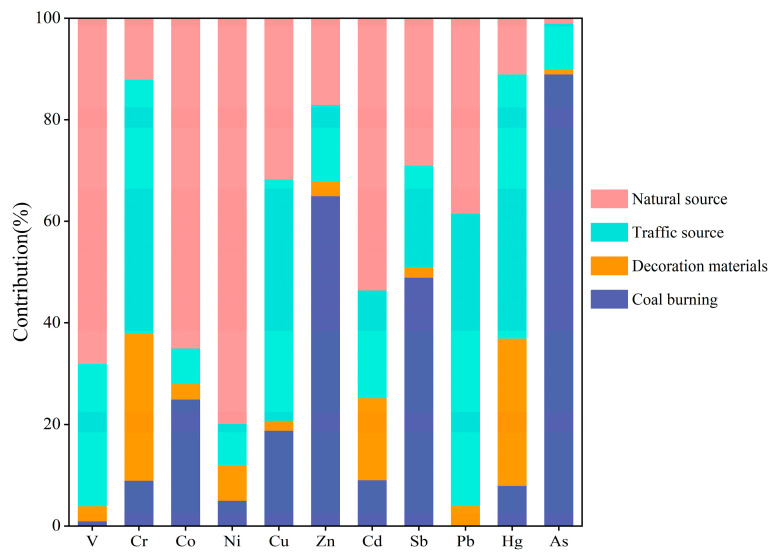
Source contribution of each factor based on APCS-MLR model.

APCS-MLR analysis showed that factor 1 was primarily contributed by V (68%), Co (65%), and Ni (79%) (Figure 7). These elements had relatively low CVs (Table 2), and their *I*_geo_ values were less than 0 (Figure 2), indicating the human influence is relatively small. Therefore, factor 1 was identified as a natural source. Factor 2 was mainly contributed by Cr (50%), Cu (48%), Pb (57%), and Hg (52%). The *I*_geo_ values of these toxic metals were relatively high (Figure 2); previous studies have verified that Pb and Hg were usually used as gasoline additives [17], Cu was widely used in brake and engine materials, and Cr was widely used to protect alloy surfaces and vehicle materials [44]. These metals can be released via vehicle exhaust and brake wear and then enriched in surface dust. Therefore, factor 2 was identified as traffic sources.

Factor 3 was characterized by Cr (29%), Cd (16%), and Hg (29%). Paint debris (powder) from UPGs’ floors was observed in samples during collection. Furthermore, Cr and Cd were commonly used as additives or pigments, and Hg was applied in building materials [17]. Therefore, factor 3 was identified as the aging of decoration materials. Factor 4 was characterized by Zn (65%), Sb (49%), and As (89%). Previous studies have verified that coal burning is one of the important sources of Sb [45], and As and Zn can also be enriched in fly ash during coal combustion [46]. Therefore, factor 4 was identified as coal combustion.

### 3.6. Influence of Essntial Conditions on Toxic Metals in UPGs’ Surface Dust

**Figure 8:**
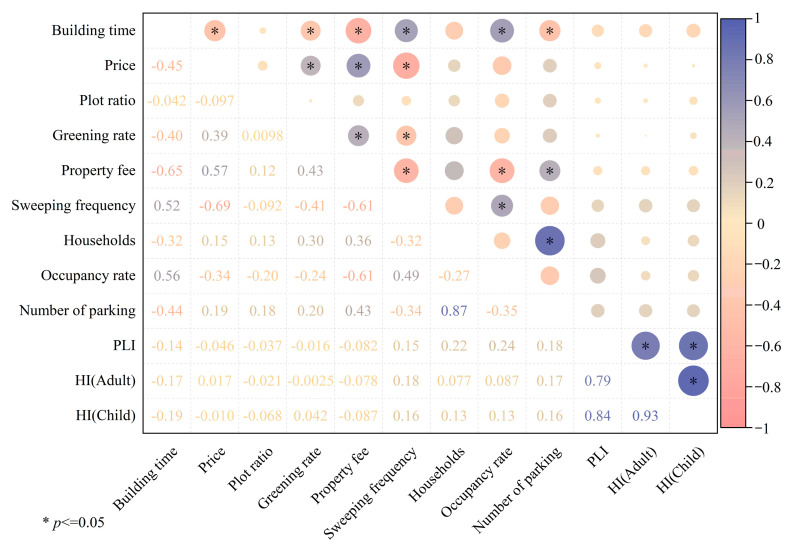
The correlation between essential conditions and pollution status and HI of toxic metals in UPGs surface dust.

The correlations between the essential conditions and the pollution status of toxic metals (PLI) and their potential health risks (HI) are shown in Figure 8. The results showed that no significant correlations were observed between these essential conditions and the PLI and HI. This result may imply that the essential conditions just have limited influence on the accumulation of toxic metals in UPGs’ surface dust. In addition, this study focused on the content of toxic metals in UPGs’ surface dust and their potential risk and disease burden to residents, and the amount of surface dust per unit area was not considered. However, the floor type and the cleaning frequency can seriously affect the amount of surface dust in per unit area. Therefore, it is more reasonable to assess the potential health risk and disease burden combined with the amount of surface dust per unit area and the content of toxic metals in these UPGs’ surface dust.

### 3.7. Limitations and Future Research

This study provides a detailed analysis of the accumulation status, potential risks, and potential sources of the toxic metals in UPGs’ surface dust. However, there are still some aspects that can be improved and further studied in the future. Firstly, continuous monitoring of toxic metal content in UPGs’ surface dust could reveal temporal or seasonal variations. Additionally, the particulate matter content in UPGs’ air is the decisive factor for the risk posed by toxic metals. Therefore, analysis of the content of particulate matter content in the air and per unit area and their toxic metal content would enable a more accurate exposure assessment. Furthermore, future studies should adopt region-, sex-, and age-specific exposure parameters to reflect varying living habits, which can improve the accuracy of risk assessment and support for more precise risk management.

## 4. Conclusions

In conclusion, several toxic metals in UPGs’ surface dust were significantly enriched, and all these surface dusts were slightly, moderately, or heavily polluted. These toxic metals posed unacceptable non-carcinogenic health risks to children, with the risk levels significantly higher than those of adults, which can be attributed to their special behaviors (crawling, hand-to-mouth) and high sensitivity. Exposure to these toxic metals leads to significant carcinogenic disease burden, with Cr being the dominant contributor. In addition, elderly populations and males exhibited higher disease susceptibility, indicating the risk posed by toxic metals in UPGs’ surface dust are significantly age- and sex-specific. Source apportionment revealed that the toxic metals in UPGs’ surface dust mainly originated from vehicle-related activities, construction and decoration materials, and coal combustion, which provided guidance for the control of the risk source. Furthermore, the essential conditions of residential areas had limited influence on toxic metals accumulation. These results suggest that more attention should be paid to the environmental safety of UPGs and reduce their potential risks and disease burden to residents.

## Figures and Tables

**Table 2:** Descriptive statistics of toxic metal contents in UPGs surface dust (mg/kg).

	Max	Min	Mean	SD	CV (%)	Background
V	185.55	37.53	68.06	33.69	49.50	84.33
Cr	565.12	48.7	126.48	100.14	79.17	46.51
Co	16.85	6.62	8.73	2.14	24.55	9.72
Ni	41.50	19.89	27.68	5.25	18.98	26.21
Cu	402.37	26.06	76.25	71.85	94.23	20.54
Zn	1274.64	98.99	287.07	225.44	78.53	77.21
Cd	2.55	0.32	0.74	0.47	64.32	0.3
Sb	23.15	1.15	4.28	3.85	89.83	1.27
Pb	943.84	22.54	172.67	202.06	117.03	24.58
Hg	0.967	0.016	0.24	0.27	112.19	0.022
As	38.873	4.060	8.66	5.96	68.77	8.04

**Table 3:** The DALYs caused by toxic metals in surface dust via different exposure pathways (per 100,000 people).

Toxic Metals		Male	Female	Total
Cr	Ingestion	1.71 × 10^0^	1.01 × 10^0^	2.71 × 10^0^
	Inhalation	1.06 × 10^−2^	6.22 × 10^−3^	1.68 × 10^−2^
	Dermal	5.13 × 10^0^	3.02 × 10^0^	8.15 × 10^0^
Ni	Ingestion	6.27 × 10^−1^	3.69 × 10^−1^	9.96 × 10^−1^
	Inhalation	9.72 × 10^−5^	5.72 × 10^−5^	1.54 × 10^−4^
	Dermal	2.25 × 10^−2^	1.32 × 10^−2^	3.57 × 10^−2^
Cd	Ingestion	1.21 × 10^−1^	7.14 × 10^−2^	1.93 × 10^−1^
	Inhalation	1.88 × 10^−5^	1.11 × 10^−5^	2.99 × 10^−5^
	Dermal	4.49 × 10^−3^	2.64 × 10^−3^	7.13 × 10^−3^
Pb	Ingestion	3.96 × 10^−2^	2.33 × 10^−2^	6.29 × 10^−2^
	Inhalation	1.23 × 10^−5^	7.22 × 10^−6^	1.95 × 10^−5^
	Dermal	3.69 × 10^−3^	2.17 × 10^−3^	5.86 × 10^−3^
As	Ingestion	3.50 × 10^−1^	2.06 × 10^−1^	5.56 × 10^−1^
	Inhalation	1.33 × 10^−4^	7.79 × 10^−5^	2.10 × 10^−4^
	Dermal	1.26 × 10^−1^	7.43 × 10^−2^	2.01 × 10^−1^
Total		8.15 × 10^0^	4.79 × 10^0^	1.29 × 10^1^

## Data Availability

The data that support the findings of this study are available from the corresponding author upon reasonable request.

## Supplementary Materials

The following supporting information can be downloaded at: https://www.mdpi.com/article/10.3390/toxics13100895/s1, Text S1: The questionnaire of apartment communities; Table S1: The information of apartment communities; Table S2: Analytical Methods and Detection Limits for Toxic Metals; Table S3: The classification of *I_geo_* and PLI; Table S4: Toxic metals contents in surface dust in other locations (mg/kg); Table S5: Influence factors of the principal factors; Figure S1: Correlation analysis between different toxic metals in UPGs surface dust.
