# Supplementary material for: Toxic Metals in Surface Dust in Underground Parking Garages: Pollution Status, Risk and Disease Burden Assessment, and Source Apportionment
Source: Toxics. 2025 Oct 19;13(10):895. doi: 10.3390/toxics13100895 (PMC12567962; doi:10.3390/toxics13100895)
Supplement: Supplementary file 1 [file toxics-13-00895-s001.zip › toxics-3894249-supplementary.pdf]

Toxic Metals in Surface Dust in Underground Parking Garages: Pollution Status, Risk and Disease Burden Assessment, and Source Apportionment

Yong Wang [1], Tong Chao [2], Qidi Li [2], Zhiqiang Jiao [2], Xinling Ruan [2,3], Yuguang Wang [4], Shiji Ge [2,*] and Yangyang Wang [2,3,*]

[1] School of Material and Chemical Engineering, Tongren University, Tongren 554300, China; wy7185299@126.com

[2] College of Geographical Sciences, Faculty of Geographical Science and Engineering, Henan University, Zhengzhou 450046, China; chaotong1229@henu.edu.cn (T.C.); liqidi9821@henu.edu.cn (Q.L.); zqjiao@henu.edu.cn (Z.J.); xlruan@henu.edu.cn (X.R.)

[3] Key Laboratory of Geospatial Technology for the Middle and Lower Yellow River Regions, Ministry of Education, Henan University, Kaifeng 475004, China

[4] School of Minerals Processing and Bioengineering, Central South University, Changsha 410083, China; ygwang@csu.edu.cn

* Correspondence: gsge@henu.edu.cn (S.G.); wangyangyangxyz@163.com (Y.W.)

Text S1. The questionnaire of apartment communities

(1) Name

____

(2) Place

____

(3) Completion time

____

(4) Price

____

(5) Plot ratio

____

(6) Greening rate

____

(7) Property management fee

____

(8) Number of houses

____

(9) Occupancy rate

____

(10) Number of parking spaces

____

(11) Garage cleaning frequency

____

(12) Ground material

____

Table S1 The information of apartment communities

Code name	Position	Build time (y)	Price (m^2)	Plot ratio	Greening rate	Property fee (m^2)	Households	Occupancy rate	Number of parking	Sweeping frequency
1	N 34.805° E 114.304°	14	7130	3.52	30	1.3	352	85	113	20
2	N 34.811° E 114.313°	5	9065	2.35	34	1.8	1200	70	1300	4
3	N 34.812° E 114.318°	14	6300	1.5	30	1	650	85	50	30
4	N 34.811° E 114.293°	3	7942	2.9	35.2	1.78	3000	35	2717	1
5	N 34.816° E 114.285°	5	10575	2.1	36	2.6	1165	45	800	1
6	N 34.815° E 114.288°	5	8542	2.1	35	1.75	2065	80	1800/85	2
7	N 34.808° E 114.282°	4	9199	1.7	30	1.89	1800	70	1500	3
8	N 34.818° E 114.286°	4	9500	1.9	45	1.8	800	70	440	1
9	N 34.814° E 114.284°	5	7905	3.6	38	1.7	1060	70	550	7
10	N 34.819° E 114.289°	4	7819	2.9	40	1.67	2000	60	1354	2
11	N 34.806° E 114.287°	4	9600	1.57	31.04	1.6	220	60	155	3
12	N 34.808° E 114.288°	8	8865	3	34	1.5	2000	63	900	3
13	N 34.807° E 114.291°	11	8340	1.6	30	1.5	759	69	350	15
14	N 34.809° E 114.291°	8	8087	3.5	40	1.9	1500	58	1276	4
15	N 34.805° E 114.283°	6	8910	3.19	45	1.6	1300	49	600	2
16	N 34.829° E 114.306°	10	6329	2.3	20	1	600	73	20	14
17	N 34.832° E 114.297°	1	7295	2.89	35	2.89	1122	20	815	1
18	N 34.809° E 114.326°	4	8845	2	34	1.6	1540	35	696	4
19	N 34.830° E 114.300°	4	9487	2	30	1.8	500	65	343	3
20	N 34.829° E 114.303°	6	5714	2.8	20	1.2	2000	70	1600	30
21	N 34.809° E 114.301°	10	5810	2.2	35	0.5	600	89	400	30
22	N 34.800° E 114.282°	6	8922	1.8	40.7	1.8	2495	85	1000	1
23	N 34.798° E 114.317°	6	7789	2	38	1.3	1200	72	200	1
24	N 34.796° E 114.293°	11	6679	1.66	40	1.5	970	74	120/100	30
25	N 34.815° E 114.312°	3	7407	2.2	40	2.5	2488	66	1800	1
26	N 34.817° E 114.311°	3	7799	1.8	40	1.68	1200	64	1000	1
27	N 34.820° E 114.316°	6	7749	2.2	40	1.88	1130	58	860	1
28	N 34.817° E 114.315°	2	9887	2.5	32	2.38	1052	60	700	1
29	N 34.816° E 114.318°	18	6020	2.5	25	0.56	822	80	100	7
30	N 34.811° E 114.310°	6	7756	2.9	25	1.25	333	80	200	7

Table S2 Analytical Methods and Detection Limits for Toxic Metals

Toxic Metals	Analytical Method	Detection Limit (DL, μg/L)
As	Atomic Fluorescence Spectrometry (AFS-3100)	0.3
Hg	Cold Vapor Generation-Atomic Fluorescence Detection (DMA-80)	0.05
V	Inductively Coupled Plasma Mass Spectrometry (ICP-MS, Thermo Fisher X2)	0.1
Cr	ICP-MS	0.2
Co	ICP-MS	0.08
Ni	ICP-MS	0.15
Cu	ICP-MS	0.2
Zn	ICP-MS	0.3
Cd	ICP-MS	0.03
Sb	ICP-MS	0.06
Pb	ICP-MS	0.12

Table S3 The classification of I_{geo} and PLI

		Classification
I_{geo}	$I_{geo} \leqslant 0$	Unpolluted
	$0 < I_{geo} \leqslant 1$	Unpolluted to moderately polluted
	$1 < I_{geo} \leqslant 2$	Moderately polluted
	$2 < I_{geo} \leqslant 3$	Moderately to highly polluted
	$3 < I_{geo} \leqslant 4$	Highly polluted
	$4 < I_{geo} \leqslant 5$	Highly to extremely high polluted
	$5 < I_{geo}$	Extremely high polluted
PLI	$0 < PLI \leqslant 1$	Clean
	$1 < PLI \leqslant 2$	Slight pollution
	$2 < PLI \leqslant 3$	Moderate pollution
	$3 < PLI$	Heavy pollution

Table S4 Toxic metals contents in surface dust in other locations (mg/kg)

	V	Cr	Co	Ni	Cu	Zn	Cd	Pb	Hg	As
Park	-	53.25	-	23.15	44.29	240.27	1.02	144.84	0.50	5.12
Bus stop	68.36	59.73	5.81	19.34	40.10	208.32	1.01	49.46	-	-
campus	-	88.36	-	29.76	47.04	328.87	1.05	68.72	-	-

Table S5 Influence factors of the principal factors

Metal	Load coefficient after rotation		
	The first principal component	The second principal component	The third principal component
V	-0.024	0.657	-0.192
Cr	0.114	0.857	0.298
Co	0.853	0.295	-0.128
Ni	0.239	0.847	-0.187
Cu	0.191	0.096	0.701
Zn	0.90	0.109	0.106
Cd	0.18	0.793	-0.21
Sb	0.936	0.077	0.194
Pb	-0.011	0.295	0.75
Hg	-0.084	0.741	0.269
As	0.965	-0.04	0.047
Variance (%)	43.05	21.69	11.78
Cumulative variance (%)	43.05	64.74	76.52

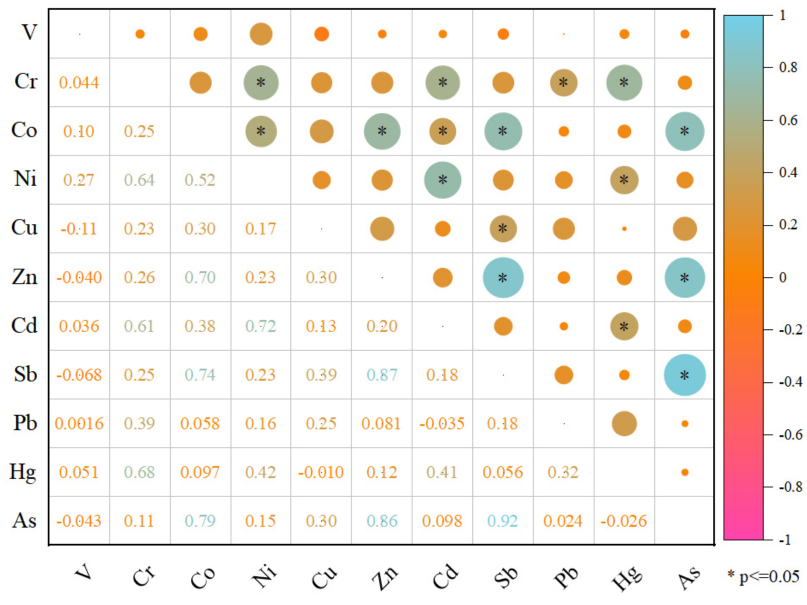

Figure S1. Correlation analysis between different toxic metals in UPGs surface dust
